# Educational intervention based on health action model to promote safe behavior of hospital service workers

**DOI:** 10.1186/s12913-023-10267-3

**Published:** 2023-11-24

**Authors:** Maryam Mohammadi, Mohammad Vahedian-sharoodi, Zahra Joghatei, Habibullah Esmaily, Hadi Tehrani

**Affiliations:** 1 Social Determinants of Health Research Center, Mashhah University of Medical Sciences, Mashhad, Iran; 2 Student Research Committee, Mashhad University of Medical Sciences, Mashhad, Iran; 3 Department of Biostatistics, School of Health, Mashhad University of Medical Sciences, Mashhad, Iran; 4 Department of Health Education and Health Promotion, School of Health, Mashhad University of Medical Sciences, Mashhad, Iran

**Keywords:** Health promotion, Health education, Healthy behavior, Health action model, Hospital personnel

## Abstract

**Background:**

Hospitals are considered to be one of the most hazardous environments to work in, and their service workers are exposed to many serious risks. So The purpose of this study was to investigate the effect of educational intervention based on the Health Action Model to promote the safe behavior of hospital service workers.

**Methods:**

In this quasi-experimental study, 45 workers in each of the control and experimental groups participated. Demographic information and data related to Health Action Model constructs were collected through a questionnaire and a checklist, immediately and three months after the intervention. Cronbach’s alpha coefficients were used to confirm the properties of the tools. Educational intervention accompanied was applied in the form of four training classes. The data were analyzed using SPSS 20 software.

**Results:**

Before the intervention, there was no significant difference between the two groups in terms of demographics and the study’s main variables. results showed significant changes in mean scores of safe behavior, Attitude, norms, belief, intention, knowledge in the experimental group three months after the intervention (P < 0.001).

**Conclusions:**

The research results show that Health Action Model educational intervention can change workers’ awareness, attitudes, norms, beliefs, and intentions toward unsafe behavior and improve their safety performance.

**Trial registration:**

IRCTID: IRCT20160619028529N7.

## Introduction

About 1.5% of all annual deaths in the world are caused by work. The amount reported in developed countries is about 40% higher than the amount in developing countries [1]. The elimination of labor force, disability and medical expenses is one of the obvious damages caused by occupational and work-related illnesses, but they also cause significant damage to economic and social development. Compared to other sectors like agriculture and industries, the health sectors are more prone to serious risks [2].

The hospital is considered the most dangerous center for providing health and treatment services in health systems, and the human resources in hospitals are exposed to various occupational hazards [3].This occupational group is at risk of all kinds of hazards such as sharp objects, disposal of hospital waste, and inhalation of formaldehyde gas and the lack of safe behavior in these people will bring irreversible consequences; training this group with the intention of safe behavior will be effective in reducing occupational accidents and improving their health [4, 5].

Despite the relatively small investment in preventive health and behavioral science, there is evidence for the effectiveness of behavior change interventions at individual, community and population levels [6, 7].

It is important to note that education alone does not play a significant role in reducing accidents, but it is necessary to pay attention to different methods and other factors influencing behavior change [8]. Theories and models in different stages of planning, implementation and evaluation of an intervention help to understand the nature of the intended health behavior and explain its dynamics and the effect of external factors on the behavior; so that the most suitable goals for programs, methods of change and evaluable results can be determined [9–11].

The health action model is one of the theories developed by Toons et al.; who presented this model in order to create a comprehensive framework of major variables affecting healthy behaviors. This theory includes two main parts of behavioral intention and factors facilitating or inhibiting behavioral intention. The first part of this model deals with the belief system, motivational, normative system and the intention of people to behave, and the second part supports the implementation of the intention by examining the obstacles [12].

In1995, Rennie described the structures of this model for use in workplace research as follows [13].

(1) Cognitive system: Workers’ basic knowledge about safety, occupational health and safe performance (2) Normative system: workplace norms and rules about safety, regulations and guidelines (3) Motivational system: the motivation of people to participate in safe practices or factors that stimulate safe behaviors in an organization. (4) Belief system: Workers’ values and beliefs about the impact and benefits of safety and safe behavior. (5) Facilitator of safe behavior: conditions and factors that lead to behaviors that cause safe performance [10, 14, 15].

## Methods

### Study design and recruitment

The present study was a quasi-experimental intervention study conducted on 142 hospital service workers in Iran. The first step involved a descriptive study to identify predictor and influence constructs of HAM on behavioral intention. For this purpose, 142 people were selected using a random sampling method. In the second step, based on similar previous studies [15, 16], taking into account the Confidence factor of 0.95% and the power of 80%, and using the sample size formula for each group, it was estimated to be equal to 40 samples. Considering of possible drop, 45 people were calculated for each group.

**Fig. 1:**
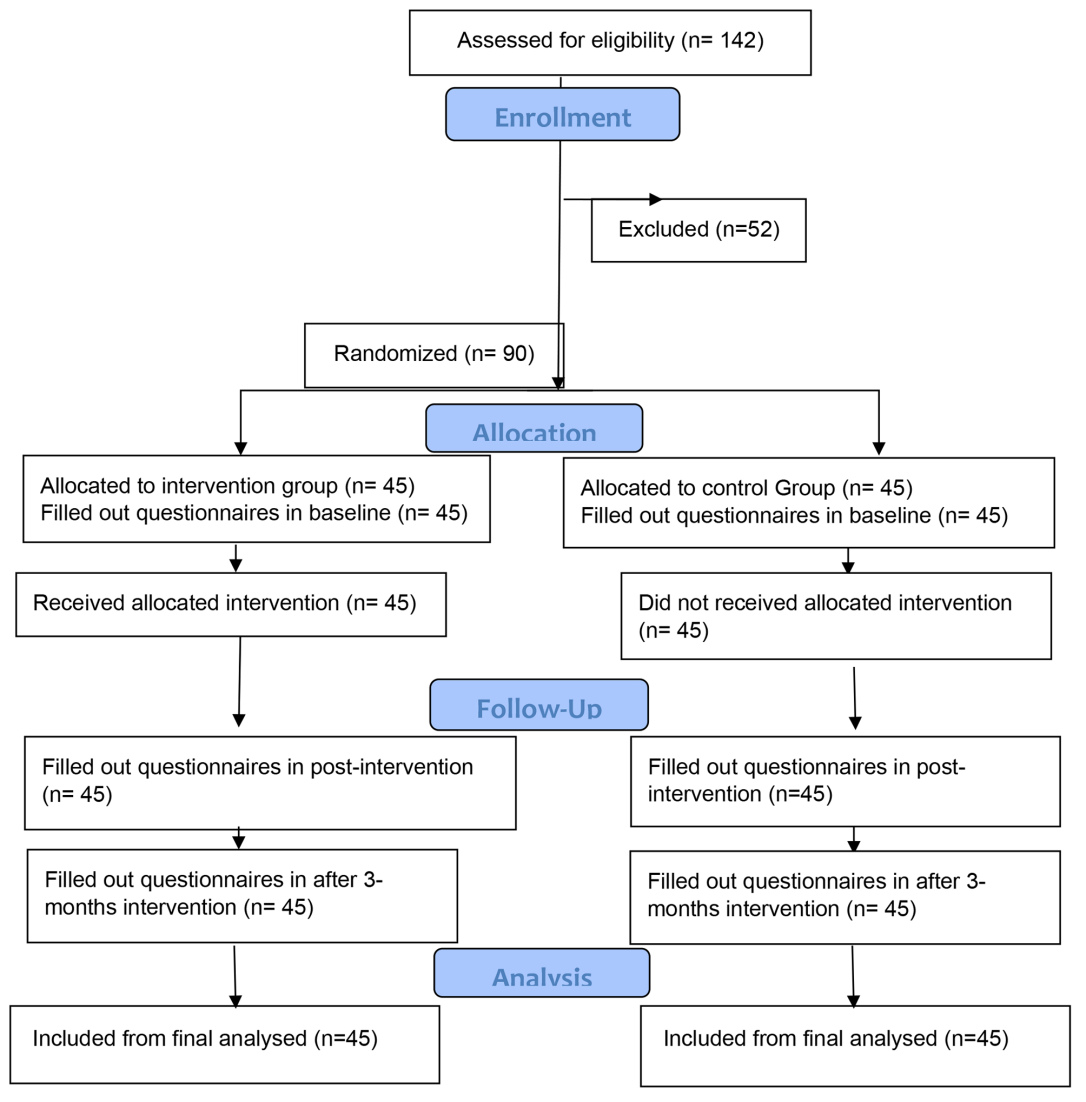
Study Design and sampling

Having at least 1 years’ experience as a hospital service workers and lack of disability were considered as inclusion criteria. Based on the items mentioned in the inclusion criteria, 52 people out of 142 participants were excluded from the study and the study was conducted on 90 people (Fig. 1).

### Intervention

The educational intervention was carried out in 6 sessions of 90 min as follows (Table 1):

It should be noted that in this research, we did not have an educational program for the control group during the study they only completed the questionnaire in the baseline, immediately and follow up stages. However, in order to comply with ethical considerations, after the end of the study, the educational package was provided to them, and those who were willing, educational sessions similar to the intervention group were held for them.

### Content validity of educational materials

In order to check the validity of the content of the educational materials prepared for the intervention, they were provided to health and occupational health education experts and the content was modified based on their opinions.

**Table 1:** educational intervention for the sessions

Session	Title	Content	Equipment	Intervention Method	Educators	Time
**1**	Knowledge	• The concept of safe behavior in the hospital • The importance of observing safe behavior while working in the hospital • Explaining the reasons for complying with safe conditions in the hospital environment	Computer, data projector Whiteboard and marker Booklets	Lecture (face to face), brainstorming group discussion	Msc in health education and occupational health	45 min
**2**	Motivation	• Interested in expressing experiences related to safe behavior while doing work for other colleagues	Computer, data projector Whiteboard and marker Booklets	Lecture (face to face), brainstorming group discussion	Msc in health education and occupational health	45 min
**3**	Belief system, Normative system & attitude	• To believe that, for his own safety, he should not use a mobile phone while doing work. • Believing in his heart that wearing rings, watches and jewelry while doing work endangers his health. • The importance of sharing the issues and problems related to safety in the hospital with the hospital management	Computer, data projector Whiteboard and marker	Brainstorming, group discussion, question and answer	Msc in health education and occupational health	45 min
**4**	Facilitators	• How to achieve safe conditions in the hospital • The importance of access to the laws, regulations and standards of health and safety conditions in the hospital	Computer, data projector Whiteboard and marker Booklets	Lecture (face to face), brainstorming group discussion	Msc in health education and occupational health	45 min
**5**	Self- efficacy	• To believe in his own ability to prevent and control accidents in the hospital environment. • To be sure that he will be able to face unexpected issues • Be aware of his inner ability to solve the problems created in the hospital environment.	Computer, data projector Whiteboard and marker	role playing, movie show, question and answer, group discussion,	Msc in health education and occupational health	45 min
**6**	Intention Behavior & Safe behaviors	• Carrying out the procedures of entering the locker room and how to change clothes and personal belongings in the presence of the instructor and students • Washing and disinfecting hands in the presence of the instructor and students • Performing the correct use of personal protective equipment in the presence of the instructor and learners • Correctly washing and brushing surgical instruments and observing safe conditions in the presence of the trainer and learners • Paying attention to the label of the disinfectant solution before using it in the presence of the instructor and students	Computer, data projector Whiteboard and marker,	role playing, movie show, question and answer	Msc in health education and occupational health	45 min

### Data collection

The HAM instrument was used for data collection. this questionnaire of the researcher made contained questions related to demographics and belief system constructs, normative systems, attitude system, self-efficacy, knowledge, behavioral intention, and facilitators. Likert scale was used to score items related to this construct. The score ranges were determined for belief system (14–70), normative system (8–40), attitude system (4–20), self-efficacy (9–45) and behavioral intention (7–35). About facilitators, the options ‘No’, ‘Somewhat’, and ‘Yes’ were assigned a score of 0 to 2, respectively.

To measure participants’ knowledge, three open-ended questions were asked about personal protective equipment needed in the workplace, risk factors, and safety recommendations. The score range for knowledge construct was in the range of 1 to 18.

To determine the validity of the questionnaires, face and content validity checks were used. In this way, the tool was given to 10 experts in occupational health and health education and health promotion, and they were asked to give their opinion on the items of the tool, regarding the appearance of the questionnaire in the form. to state completely suitably, appropriate and inappropriate and after obtaining opinions, the necessary changes were applied and content validity was also confirmed by checking CVI = 079. In this research, Cronbach’s alpha method was used to determine the reliability of the questionnaire, in this way. The questionnaire was completed by 50 people from the hospital staff, and the alpha of 0.72 for the whole instrument was confirmed based on Robert’s opinion [17].

### Questionnaire completion

It should be noted that the completion of the questionnaire was done by people in person and within 20–30 min, and if there was any ambiguity in a question, it was explained to them.

### Data analysis

To analyze the data, the Kolmogorov-Smirinov test was used to check the normality of the variables. Then, due to the nonparametric of the data, Mann-Whitney tests were used for variables in independent groups and Friedman’s test was used for variables in dependent groups. For analyze the demographic variables in qualitative variables, mean, standard deviation and chi-score test were used, also frequency and percentage were used for quantitative variables.

## Results

In this research, 142 service workers of Bojnord hospitals were included in the study, 51.4% of the participants were male and 48.6% were female. Also, the majority of participants (61.3%) were married and the average age was 36.75 ± 7.1 years. Regarding the level of education, 53.5% of the participants had less than a diploma and 45.5% had a diploma or higher. Statistical analysis showed that the two groups were homogeneous in terms of demographic variables and did not have statistically significant differences (p < 0.05) (Table 2).

**Table 2:** demographic variables in the research units

Variable	Variable levels	Intervention group		control group		Test result
		Number	Percent	Number	Percent	
**Gender**	Male	23	51.1	23	51.1	χ^2^ = 0.01 p-value = 0.99
	Female	22	48.9	22	48.9	
**Marital status**	Single	13	28.9	14	13.1	χ^2^ = 0.05 p-value = 0.81
	Married	32	71.1	31	68.9	
**Education**	Elementary	5	11.1	5	11.1	χ2 = 0.05 p-value = 0.77
	Middle school	12	26.67	15	33.3	
	Diploma	28	55.6	25	62.2	
**Employment Status**	Corporate	22	48.9	23	51.1	χ2 = 0.04 p-value = 0.83
	Contractual	23	51.1	22	48.9	
		**Mean**	**SD**	**Mean**	**SD**	
**Age**		37.26	0.12	37.35	0.23	z = -0.02 p-value = 0.97
**work experience**		8.1	24.5	8.2	27.53	z = -0.21 p-value = 0.83

The results of this research showed that before the implementation of the educational intervention, the average knowledge score in the intervention and control groups was not significant (P = 0.44), but immediately and three months after the intervention, there was a significant difference between the groups (P < 0.001).

In the context of comparing the average scores of the belief, the results showed that there was a significant difference between the average scores of the intervention group after the intervention and the scores of the control group. (p < 0.05) before the educational intervention, the average score of the belief structure in the intervention group was 53.02 ± 6.61 and in the control group 51.97 ± 6.7, there was no significant difference (P = 0.45). Also, before the educational intervention, the average score of the normative structure in the intervention group was 29.4 ± 4.52 and in the control group was 29 ± 4.64, and there was no significant difference between these two groups. (P = 0.83) immediately and three months after the intervention, there was a significant difference in the norm structure between the groups. (p > 0.001) before, immediately and three months after the intervention, a significant difference was observed in the intervention group (p > 0.001), but there was no significant difference in the control group (p = 0.18). Before the educational intervention, the average score of facilitating factors in the intervention group was 10.84 ± 2.53 and in the control group was 10.17 ± 2.91. And there was no significant difference between these two groups. (P = 0.76) immediately and three months after the intervention, there was a significant difference between the groups. (P < 0.001) (Table 3).

Also, before the educational intervention, the average score of behavioral intention in the intervention group was 30.42 ± 2.83 and in the control group was 30.53 ± 2.88 and there was no significant difference between these two groups. (P = 0.87) but immediately and three months after the intervention, there was a significant difference between the groups. (P < 0.001) Before the educational intervention, the average score of safety behaviors in the intervention group was 13.13 ± 1.71 and in the control group was 13.24 ± 1.79, and there was no significant difference between these two groups. (P = 0.78) Immediately and three months after the intervention, there was a significant difference in safety behaviors between the groups. Also, changes in safety behaviors immediately and three months later compared to before the intervention between the intervention group and the control were significant. (p > 0.001) before, immediately and three months after the intervention in the group Intervention (p < 0.001) significant difference was observed, but there is a significant difference in the control group there was none (p = 0.5).

Examining the average self-efficacy scores of the servants before the educational intervention was 28.62 ± 2.49 in the intervention group and 28.42 ± 2.63 in the control group, and there was no significant difference between these two groups (P = 0.62). After the training, the average scores of the intervention group’s servants increased to 33.42 ± 2.14 three months later, which was statistically significant (p > 0.05) but no noticeable change was seen in the control group.

**Table 3:** **Score of constructs of HAM before, immediately and three months after the intervention in the control and intervention groups**

Study time		Before the intervention (Mean ± SD)	Immediately after the intervention Mean ± SD	Three months after the intervention (Mean ± SD)	p-value
Structures Of the model	Groups of study				
**Belief**	intervention	53.02 ± 6.61	62.71 ± 2.38	52.71 ± 4.42	p < 0.001
	Control	51.97 ± 6.7	52.42 ± 5.85	47.77 ± 6.55	p < 0.001
	p-value	p = 0.45	p < 0.001	p < 0.001	
**Norm**	intervention	29.4 ± 4.52	29.04 ± 4.32	29.24 ± 4.25	p = 0.18
	control	29 ± 4.64	37.75 ± 2.24	35.15 ± 3.43	p < 0.001
	p-value	p-value = 0.83	p-value < 0.001	p-value < 0.001	
**Attitude**	intervention	12.55 ± 2.37	17.31 ± 1.98	16.06 ± 2.6	p < 0.001
	Control	12.55 ± 2.4	12.46 ± 2.41	12.48 ± 2.36	p = 0.86
	p-value	p = 0.86	p < 0.001	p < 0.001	
**Intention behavior**	control	30.53 ± 2.88	30.24 ± 3.008	30.11 ± 2.85	p = 0.02
	intervention	30.42 ± 2.83	34 ± 1.02	33.24 ± 1.44	p < 0.001
	p-value	p = 0.87	p < 0.001	p < 0.001	
**Motivation**	intervention	22.13 ± 1.45	30 ± 1.88	27.08 ± 1.72	p = 0.003
	Control	21.93 ± 1.38	21.53 ± 1.57	21.48 ± 1.51	p < 0.001
	p-value	p = 0.55	p < 0.001	p < 0.001	
**Self-efficacy**	Control	28.42 ± 2.63	28.02 ± 2.54	28.2 ± 2.54	p = 0.02
	intervention	28.62 ± 2.46	33.68 ± 1.34	33.42 ± 2.14	p < 0.001
	p-value	p-value = 0.62	p-value < 0.001	p-value < 0.001	
**Knowledge**	intervention	39.57 ± 2.44	43.35 ± 1.33	41.97 ± 1.68	p < 0.001
	Control	39.93 ± 2.56	39.68 ± 2.48	39.46 ± 2.37	pv0.006
	p-value	p-value = 0.44	p-value < 0.001	p-value < 0.001	
**Facilitator of intention** **behavioral**	intervention	10.84 ± 2.53	14.06 ± 1	12.48 ± 1.03	p < 0.001
	Control	10.17 ± 2.91	10.48 ± 2.8	10.64 ± 2.97	p = 0.02
	p-value	p = 0.76	p < 0.001	p = 0.001	
**Safe behaviors**	intervention	13.13 ± 1.71	12.33 ± 1.7	12.53 ± 1.68	p < 0.001
	Control	13.24 ± 1.79	13.33 ± 1.77	13.35 ± 1.76	p = 0.5
	p-value	p = 0.78	p = 0.008	pv0.03	

## Discussion

### General description of the purpose of the study

The aim of this study was to investigate the effect of education based on Health Action Model structures on the promotion of healthy behavior, which can be seen in Table 3 based on individual structures at different times of the study. For this purpose, 142 service workers of Bojnord hospitals were included in the study. Questionnaires were completed for all the participants in the study and the factors and the structures influencing safe behavior were determined, and the results showed that demographic variables had no significant relationship with safe behaviors, only a significant relationship was seen between gender and safe behaviors so that the mean score of safe behaviors It was higher in women than in men.

### Discussion about model structures

#### Attitude

The results of this study in relation to model structures showed in the field of attitude paying attention to attitude as the only factor cannot be considered a valid and generalizable predictor for occupational accidents, although most safety studies focus on people’s attitudes and the change of attitude toward Safety is the main factor affecting the behavior of a person, but other factors affect the effect of attitude on behavior, which has received less attention. Attitude change can have a major impact on injuries through the influence of social norms [18]. In the study of Clark et al., it was also stated that people’s beliefs are more predictive than their attitudes in the field of safety and occupational accidents [19].

#### Belief system

Also, the results of this research showed the positive effect of educational intervention on improving the level of people’s beliefs. which was in line with the results of the study by Fan in China [20] Also, these results were confirmed in the study by Mazaheri et al [15] .

The significant change in the beliefs of the workers in the intervention group was mainly related to the increase of their awareness in the field of the desired behavior and the positive experiences of people after performing safe behaviors, and naturally, the holding of training classes and group discussion was effective in changing the beliefs of the workers. Because in group classes, a supportive atmosphere is created for individuals, and participation in these classes will have psychological benefits for individuals, and during that group members provide suggestions to deal with specific problems [21, 22].

#### Normative structure

The results of this study in the field of normative structure score were consistent with the study of Mazaheri and colleagues in Isfahan [15]. Studies conducted in this field emphasize the impact of norms and effective people including managers on safety performance, safety studies have shown that there is a very important and special relationship between managers and workgroup members. This communication encourages and strengthens safe behaviors [23].

Management plays an important role in the adequacy and efficiency of safety programs. Managers should actively implement people’s ideas in the field of safety, formulate safety regulations in an applicable manner and supervise their implementation, and allocate sufficient resources to safety, deal with suggestions and complaints related to safety as soon as possible. attend safety meetings and pay attention to safety and health training and visit the workplace regularly and follow safety regulations more than others [24, 25].

Another very important and effective variable in the field of norms is the reaction of colleagues toward safety. The attitude of workers in the field of safety is affected by the norms of the group of colleagues. Unfortunately, most of the time workers see their co-workers doing risky behaviors but they don’t talk about it and fail to report it even when they know they should.

Studies conducted in multiple organizations have shown that 90% of people believe that workers should warn others of unsafe practices, however, only 60% of people do it. There is a deep gap between people’s values ​​ (need to warn others) and actual behaviors (warning). Researchers in organized interviews with workers, about the reason for this question, have done, most people answered that giving safety-related feedback causes hostility between people or that giving safety feedback is not their job; Or most people think that they are not competent enough to give safety feedback and either they don’t want or they don’t want to disrespect colleagues who are more experienced than themselves [24].

### Facilitators safe behavior

The facilitating factors of behavioral intention immediately and three months later compared to before the intervention was significant between the intervention and control group. showed similar results [12, 16].

Safety education in hospitals is not a simple process and requires skillful interaction with other people [12] Therefore, it is necessary for workers at all levels of the organization to be sincerely and continuously praised for safe behaviors [26]. One of the most difficult Ways to improve safety culture and prevent accidents is to maximize safety-related communication throughout the organization [12].

Therefore, providing corrective feedback, honest encouragement, or appreciation for a job well done can increase the understanding of individual freedom and empower people [12]. Other measures to facilitate safe behavior include providing better personal protective equipment and better distribution of this equipment. Personal protective equipment creates a barrier between workers and hazards in the workplace. Therefore, it is necessary to provide personal protective equipment and related equipment to the workers and to ensure that this equipment is suitable for the type of work and risks and that they are available in sufficient numbers and are provided to the workers free of charge. They are regularly reviewed, and they are kept in the right place and in a good way, it is also necessary to teach the workers the correct way to use these devices [16].

#### Behavioral intention

The results of this research in the field of the effect of training on the behavioral intention of the servants regarding the promotion of safe behaviors before, immediately, and three months after the intervention in the two experimental and control groups showed a significant difference between the groups. (P < 0.001) The results of the study by Mohammadi Zaidi et al. showed that the average score of behavioral intention in the group under study changed significantly in the third month of follow-up [23].

Also, the results of Mazaheri and colleagues [15] showed that the difference in the mean scores of safe behavior intention in the workers of the experimental and control groups was significant after the intervention, which was in line with the results of the present study. This significant change can show the positive effect of the intervention in promoting the intention of safe behavior.

#### Safe behaviors

Another goal of this research was to determine the effect of training on the safe behaviors of the servants regarding the promotion of safe behaviors before, immediately, and three months after the intervention in two experimental and control groups. The results showed changes in safety behaviors before, immediately and three months after the intervention in the group Intervention (p < 0.001) significant difference was observed; But there is a significant difference in the control group (p = 0.5) In this regard, the study by Mohammadi et al [27] showed that despite the significant difference between the intervention and control groups, the safety behavior score was lower than the present study and the reason for this difference could be the program general education held by the management system of hospitals.

#### Self-efficacy

Examining the average scores of the self-efficacy of the servants before the educational intervention in the intervention and control groups showed a significant difference between these two groups immediately and after the intervention.

In the usual training to change the behavior of workers, less attention is paid to self-efficacy, while attention to this variable is essential in creating and promoting safe behaviors. If a person does not consider himself efficient to perform a task, he will naturally perform that task (behavior) with less probability. Because perceived self-efficacy in the context of behavior is considered a prelude to performing that pressure [25].

Therefore, self-efficacy was considered one of the influencing variables, and in the intervention program, self-efficacy creation resources were provided for them, which included substitute experiences, repetition and skill practice, verbal persuasion, and the feasibility of the skill from the workers’ point of view.

In addition to reporting workplace hazards, involving workers in workplace safety and health programs is one of the effective ways to control unsafe behaviors and motivate them to perform safe behaviors through training [12]. It can be said that the success of this intervention in promoting Safe behavior and reducing the number of accidents was partly due to the collaborative method (group discussion, practical exercises, identifying and prioritizing risks, and providing corrective action).

Another reason for the remarkable success of this intervention was the use of safe behavior patterns. In Montegrey’s study, which used the safe behavior model as a four-stick for safety training programs, it was observed that this model is well able to determine and describe the variables affecting safety behaviors in the food industry [12].

Therefore, the use of behavior change patterns and the design of interventions based on these patterns, along with collaborative methods, can lead to more successful interventions.

#### Application of research results

In working environments and decision-making, the use of research evidence and documentation leads to the improvement of the health of the workforce [28].

Although the behavior modification program is one of the valuable components of safety management, it should be noted that this program replaces management strategies such as safe designs and risk control.

Unsafe actions are only a part of unsafe conditions in the workplace. Therefore, the safety management system should prepare various programs aimed at identifying and eliminating these conditions.

### Limitations

One of the limitations of the present research is the possibility of answering the questions of the questionnaire under the influence of the personality, mental state, beliefs, and opinions of the servants and the fear of giving real answers (especially the servants of the company who did not have much job security and answered extremely conservatively) They gave). And there was also the possibility of holding training classes interfering with the work shifts of the personnel.

## Conclusion

Since the workers of any organization are considered the most valuable assets of that organization and healthy workers are a powerful tool in the hands of management to achieve organizational goals and sustainable development, therefore preventing accidents is considered one of the valuable missions of an organization. Because the accident is always lurking and you should always be ready to prevent and deal with them, all workers must be responsible for their safety and accept their health and that of others achieving safety and the highest level of health is considered valuable from the point of view of the organization and all its members.

On the other hand, considering that behavior is a complex process and its change requires a documented plan, but despite this, the present study by holding collaborative training sessions and collecting data in two stages immediately after the intervention and after three months. It showed that training with an emphasis on HAM structures has made changes in the way the intervention group works to promote safe behaviors. Maybe it is possible to achieve more success by spending a long time following up and maintaining it. Also, considering the success of the results of this study and the importance of preventing accidents caused by work injuries, it is hoped that the results of this research will be fruitful in all hospitals and prepare the ground for improving the safety culture in hospitals. In addition, due to the low cost of preventive activities from occupational accidents compared to therapeutic activities in this field, it seems necessary to generalize such educational programs and expand them to change related behaviors.

In the end, the researchers hope that the officials and those involved in the safety of the hospitals, according to the results of the studies, will adopt appropriate policies to improve in-service training and promote the participation of the servants in the processes related to safety and ultimately safe behaviors in the workplace. compile The results of this study can be a basis for future research in the field of applying methods to create safe behavior.

And the researchers suggest that in future research, the pattern of safe behavior in nurses and other occupational categories working in the hospital with other methods (non-questionnaire methods such as interviews) also, be measured.

## Data Availability

Data will be provided by the corresponding author upon request.
